# Diagnostic evaluation of IgM ELISA and IgM Immunofluorescence assay for the diagnosis of Acute Scrub Typhus in central Nepal

**DOI:** 10.1186/s12879-020-4861-y

**Published:** 2020-02-13

**Authors:** Rajendra Gautam, Keshab Parajuli, Tshokey Tshokey, John Stenos, Jeevan Bahadur Sherchand

**Affiliations:** 1 Department of Microbiology, Maharajgunj Medical Campus, Institute of Medicine, Kathmandu, Nepal; 2 Australian rickettsial reference laboratory, Geelong, Victoria Australia

**Keywords:** Scrub typhus, ELISA, *Orientia tsutsugamushi*, Nepal

## Abstract

**Background:**

Scrub typhus is an acute febrile illness caused by the obligate intracellular bacterium, *Orientia tsutsugamushi*. Immunochromatography (ICT) and IgM ELISA are two of the routinely employed antibody based assays for diagnosis of Scrub typhus fever in Nepal, although the recommended gold standard diagnostic test is IgM Immunofluorescence assay (IFA). This study evaluated InBios Scrub Typhus Detect™ Immunoglobulin M (IgM) ELISA and IgM Immunofluorescence assays in single serum sample at the time of admission.

**Method:**

Study participants (1585 suspected cases), were enrolled based on acute febrile illness with suspected scrub typhus cases in central Nepal. Blood sample was collected from the suspected patients of scrub typhus, presenting with acute febrile illness. IgM antibody to *Orientia tsusugamushi* was detected by using Scrub Typhus Detect™ Kit and an in-house IgM IFA. The IFA assay was performed with the Gilliam, Karp, Kato strains and *O. chuto* antigens following the ARRL protocol.

**Result:**

Statistical analysis of IgM ELISA results when compared to reference test, IgM IFA results demonstrated the following characteristics, sensitivity 84.0% (95%CI: 79.73–87.68%), specificity 94.82% (95% CI: 93.43–95.99%), positive likelihood ratio 16.21% (95% CI: 12.71–20.67%), negative likelihood ratio 0.17% (95% CI: 0.13–0.21%), disease prevalence 22.08% (95% CI: 20.06 -24.21%), positive predictive value 82.12% (95% CI: 78.28–85.42%) and negative predictive value 95.44% (95% CI: 94.27–96.38%) respectively.

**Conclusion:**

Although IgM IFA is considered the gold standard test for the diagnosis of scrub typhus cases, it is relatively expensive, requires trained personal and a microscope with fluorescence filters. Scrub typhus IgM ELISA may be the best alternative test and possible viable option for resource limited endemic countries like Nepal.

## Background

Scrub typhus is an acute febrile illness caused by the obligate intracellular bacteria *Orientia tsutsugamushi*. Scrub typhus is an arthropod-borne illness with the larval stage of several species of trombiculid mites called as chiggers; act as vectors for the transmission of the disease to the human beings. The species of the genus *Leptotrombidium,* particularly *leptotrombidium deliense* is considered to be primary cause of disease transmission in most countries [1]. Humans become infected with *Orientia tsutsugamushi* via the bite of an infected chigger, which act as both the vector and reservoir of *Orientia tsutsugamushi*.

Traditionally, the scrub typhus was considered to be endemic in the part of world described by ‘tsutsugamushi triangle’ which consists the northern most point in Korea, Japan and the far eastern Russia in the north, reaching to tropical northern Australia in the south and Pakistan and Afghanistan in the west [2]. However, in recent years, serological evidence shows its presence outside the tsutsugamushi triangle [3].

Nepal is considered as an endemic area of tsutsugamushi triangle and the outbreak of this disease is reported from different districts [4]. Early detection is difficult due to similarity of symptoms with other acute febrile illness such as malaria, dengue, leptospirosis, viral hemorrhagic fevers and enteric fever. Rapid and accurate diagnosis is essential for proper treatment and prevention of lethal complications. Various serological methods are available for the diagnosis of scrub typhus such as Weil-Felix test, Immunochromatographic test (ICT), Enzyme linked immunosorbent assay (ELISA), Immunofluorescence assay (IFA), Indirect immunoperoxidase assay. Molecular techniques (Real time and conventional PCR) and isolation of the organism *O. tsutsugamushi* from blood and eschar of the patient are also utilized, however culture is not commonly utilised.

Antibody based diagnostic assays are important for the diagnosis of scrub typhus in resource limited countries like Nepal. Although the gold standard test for diagnosis of acute scrub typhus is IgM IFA [5, 6], ICT and IgM ELISA are routinely employed in Nepal. The scrub typhus IgM ELISA was first developed after the purification of the antigens derived from the host cells [7]. An assay utilizing the *O. tsutsugamushi-*specific recombinant 56-kDa antigen is now available as a commercial ELISA kit.

Despite the wide use of ICT and IgM ELISA, data on their performance and efficacy in resource limited countries like Nepal, is not widely available. Hence this study was designed to evaluate the performance of the ELISA in comparison with an in-house IgM IFA in an acute serum sample taken at the time of admission.

## Methods

### Samples

A prospective study was conducted among hospitalized acute febrile illness patients with suspected scrub typhus in central Nepal for a period of one year, April 2017 to March 2018. Cases of uncharacterized acute fever for more than 4 days were included in the study after excluding the confirmed cases of other febrile illness caused by malaria, dengue, leptospirosis, and enteric fever. Single blood sample were collected from this subset of acute febrile illness patients that were hospitalized. The blood samples were centrifuged at 3000 rpm for 5 min to separate the serum. Serum samples were stored at − 80 °C until they were analyzed.

### Scrub typhus detect™ IgM ELISA

IgM antibody to *Orientia tsutsugamushi* was detected by using Scrub Typhus Detect™ Kit, InBios International, USA containing the recombinant p56kDa type specific antigens of *Orientia tsutsugamushi* Karp, Kato, Gilliam and TA 716 strains according to the manufacturer’s instruction. Optical density was measured by HumaReader HS, ELISA reader, optical densities (ODs) > 0.50 was considered positive. The cut-off was calculated following recommendations for determining the endemic cut-off titre in the kit protocol. The cut-off calculated from healthy volunteer was mean OD (0.23) + 3 standard deviation (0.09) =0.50. We proposed a cut-off OD value of > 0.50 for chitwan and surrounding region based on our finding.

### IgM immunofluorescence assay

Antibodies against Scrub Typhus Group were tested using *Orientia tsutsugamushi* (Gilliam, Karp, Kato) strains and *Orientia chuto* antigens. The antigens were prepared in the Australian Rickettsial Reference Laboratory, Geelong, Australia by culturing the organism in L929 cell line and RPMI media (Invitrogen) supplemented with 5% fetal bovine serum. Individual antigens were coated onto rickettsial screening slides containing 40 individual wells, air dried and fixed in acetone. Serum samples were diluted 1:128 in 2% casein buffer and spotted onto the slide in duplicate and incubated at 37 °C for 40 min in a moist chamber to allow for the binding of antigen and antibody. With each slide tested, positive and negative controls were included. Slides were washed 3 times in PBS and dried. An anti-human FITC labeled IgM conjugate was then added and slides incubated at 37 °C for 40 min in a moist chamber. Slides were washed once more, air dried, mounted and observed under the fluorescent microscope. Positive results are indicated when fluorescence intensity was equal to or greater than the positive control. The diagnostic cutoff > 1:128 was considered positive which was derived after testing the serum samples of healthy controls from that particular region. Negative results were reported when the sera didn’t fluoresce at a dilution of 1:128. Positive serum samples were serially titrated 1:128, 1:256, 1:512, 1:1024, 1:2048, 1:4096, 1:8192, 1:16384 etc. to end point titers with individual antigens.

### Quality control

Positive and negative controls were included with each slide that if either failed in a screening or titration slide, tests were repeated. In instances of continuation of assay failure both the antibody and antigen controls were re-titrated to see if there had been a shift in the antibody endpoint or if the antigen had lost its reactivity. Whenever necessary fresh controls and antigens were optimised prior to repeating of the assay with the specimens.

### Statistical analysis

The collected data were entered in Epi info 3.5 from CDC and exported to IBM SPSS version 16.0 (SPSS Inc. Chicago, USA). The sensitivities, specificities, positive predictive values, negative predictive values of the serological tests were calculated using MedCalc for windows, version 18.11.3 (MedCalc, Software, Ostend, Belgium). STARD 2015 guidelines for reporting diagnostic accuracy studies was strictly followed [8].

## Results

**Fig. 1:**
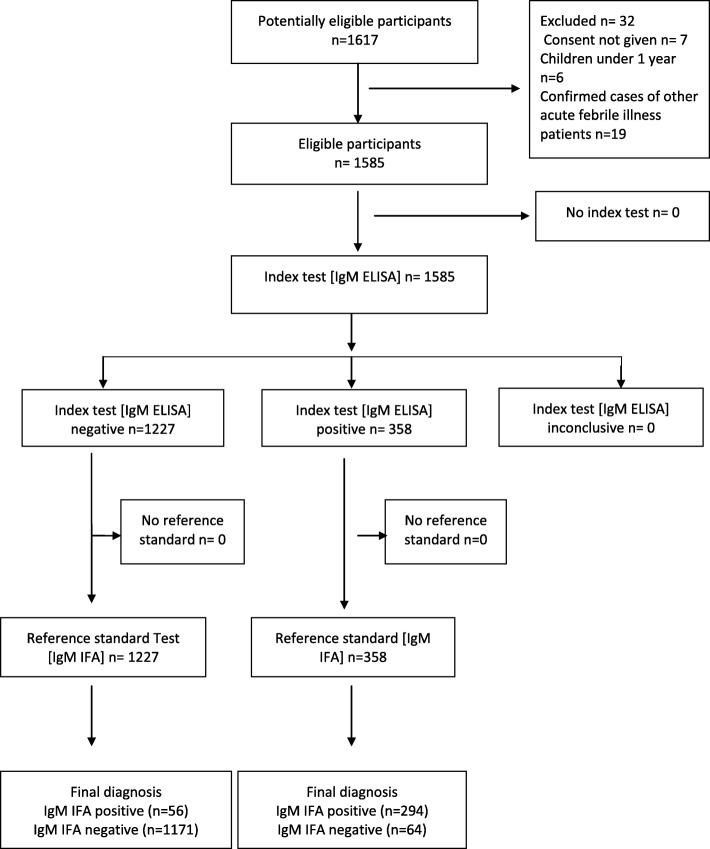
Standard for Reporting Diagnostic Accuracy (STARD) Flow Chart of the suspected Scrub typhus cases enrolled in the study

**Fig. 2:**
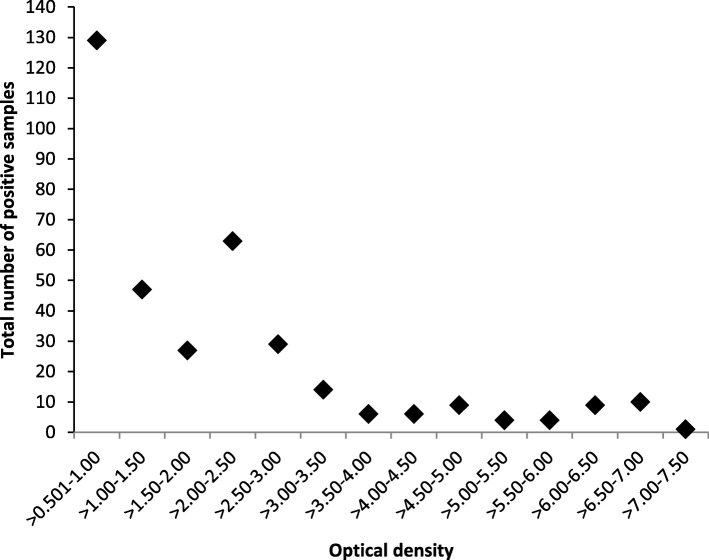
OD Values for *Orientia tsutsugamushi* IgM ELISA Positive samples (n=358)

**Fig. 3:**
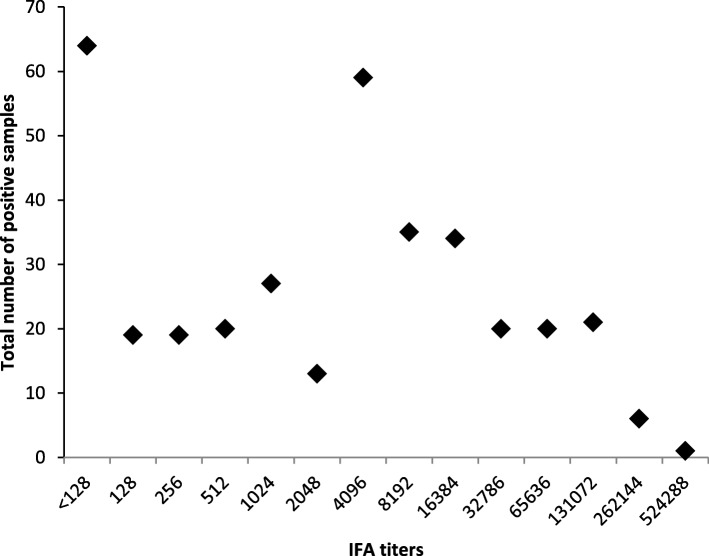
IgM IFA reciprocal titer for *orientia tsutsugamushi* IgM ELISA positive samples (*n* = 358)

Standard for Reporting Diagnostic Accuracy (STARD) flow chart of suspected scrub typhus cases enrolled in the study is given in Fig. 1. Out of clinically suspected 1585 cases 358 (22.58%) were IgM ELISA positive, OD Values for *Orientia tsutsugamushi* IgM ELISA Positive samples are summarized in Fig. 2, of these 294 were also positive by IgM IFA. Among these 358 IgM ELISA positives, the mean age of the patients was 29.7 years with female preponderance (61.7%), fever was the most common (100%) clinical characteristic followed by nausea (50.6%) with thrombocytopenia in 74.09%, presence of eschar was observed in 3.1% patients. The median number of days of fever prior to hospitalization was 7. The IgM IFA endpoint titers for *Orientia tsutsugamushi* antigens IgM ELISA positive samples are listed in Fig. 3. Among the 1227 IgM ELISA negative samples 56 were positive by IgM IFA. Comparison of IgM ELISA and IgM IFA results are summarized in Table 1.

**Table 1:** Comparison of IgM ELISA and IgM IFA test (*n* = 1585)

Description		Immunofluorescence IgM Test		
		Positive	Negative	Total
ELISA IgM	Positive	294 (18.54%)	64 (4.03%)	358 (22.58%)
	Negative	56 (3.53%)	1171 (74.34%)	1227(77.41%)
	Total	350 (22.08%)	1235 (77.91%)	1585 (100%)

Statistical analysis of ELISA IgM results when compare to IgM IFA results demonstrated the following characteristics, sensitivity 84.0% (95% CI: 79.73–87.68%), specificity 94.82% (95% CI: 93.43–95.99%), positive likelihood ratio 16.21% (95% CI: 12.71–20.67%), negative likelihood ratio 0.17% (95% CI: 0.13–0.21%), disease prevalence 22.08% (95% CI: 20.06 -24.21%), positive predictive value 82.12% (95% CI: 78.28–85.42%), negative predictive value 95.44% (95% CI: 94.27–96.38%) respectively. Calculation of statistical values of IgM ELISA test compared with IgM IFA are summarized in Table 2.

**Table 2:** Calculation of statistical value of IgM ELISA test compared with IgM Immunofluorescence test

Statistic	Value (%)	95% CI
Sensitivity	84.00	79.73–87.68
Specificity	94.82	93.43–95.99
Positive likelihood ratio	16.21	12.71–20.67
Negative likelihood ratio	0.17	0.13–0.21
Disease prevalence	22.08	20.06–24.21
Positive predictive value	82.12	78.28–85.42
Negative predictive value	95.44	94.27–96.38

## Discussion

Scrub typhus is an endemic/reemerging acute febrile infectious disease prevalent in Nepal. The diagnosis of scrub typhus was generally done by clinical presentation and history reporting prior to its serological diagnosis. The common clinical manifestations of other acute febrile illness make it difficult for its clinical diagnosis. The mortality rate varies from 0 to 30% if left untreated in the endemic areas [9].

In this study we compared the results of the Scrub Typhus Detect™ IgM ELISA against the gold standard IgM IFA.

The overall sensitivity and specificity of the IgM ELISA was 84.0% (95% CI: 79.73–87.68%) and 94.82% (CI: 93.43–95.99%) respectively in our study. Various studies have demonstrated a similar performance, with sensitivities in the range of 85 to 93% and specificities 94 to 97.5% [10–12].The diagnostic cut-off OD in this study varied from > 0.50–7.50. Sensitivity of IgM ELISAs varies from 70 to 100% according to various scientific reports [13–15].

InBios IgM ELISA is a sensitive and specific test and could be substitute for the IgM IFA in resource limited countries like Nepal. ELISA is comparatively cheaper and easier test with high throughput of the specimens.

Indirect immunofluorescence assay (IFA) is considered to be the gold standard test for the diagnosis of scrub typhus [16], in which the patients serum containing the antibodies to *Orientia tsutsugamushi* are mixed to antigen on a slide, then detected using a fluorescently labeled anti-human antibody. Karp, Kato and Gilliam serotypes are the most commonly used antigens [16], local serotypes are also included in some area [17]. Among 1585 samples 350 (22.08%) were positive IgM IFA in our study. End point titer ranged from > 1:128–1:524,288. High titer observed in our study may be due to active phase of the disease. The sensitivity of IFA varies from 70 to 100% and specificity varies from 84 to 100% according to the various scientific publications [11, 14, 15]. Diagnostic cut-off titers of IFA vary from 1:10–1:400, which may lead to confusion in its use in the endemic area [16].

Local cut off titer must be determined in the endemic countries based on the seroprevalence rate in the healthy population while using IFA and ELISA, which enables to distinguish the acute infection and previous exposure if only single serum sample is available [16]. Although IFA is loosely considered the gold standard diagnostic test, it is an imperfect reference test, particularly when using only acute samples for diagnosis. IFA has additional limitations as this depends on the microscopist who reads the slides and determines the end-point titer. There might be inter- and intra-operator variability so microscopists must be supervised by more experienced laboratory professional and undergo several months of training before their results can be considered reliable [18]. Requirements of a trained personal, fluorescent microscope, validation of appropriate diagnostic cut offs and relatively high cost is the main drawback of the IFA. Even with all these negatives, IFA stands out as a far superior test in scrub typhus diagnosis.

The various limitations of this study include the collection of single serum specimens for the detection of IgM antibodies during the acute phase of the disease. A rise in the antibody titer in paired sera could not be detected in our study. The circulating strains of Orientia species in Nepal are not known so antigens used in the serological assays *Orientia tsutsugamushi* Karp, Kato, Gilliam, TA 716 and *Orientia chuto* may not be representative of the infecting Orientia from this region which may lead to lower sensitivity of both ELISA and IFA used. The antigens used for ELISA test (TA716) and IFA (*O. Chuto*) may lead to minor inaccuracies in the result and validation between the IFA and ELISA.

## Conclusion

Scrub typhus is the common acute febrile illness present in our country Nepal. Although IgM IFA is considered the gold standard test for the diagnosis of scrub typhus cases, it requires trained personal and fluorescent microscope. IFA is gold standard test which need to be utilized for all research purposes. It may be difficult to use this method for the routine diagnosis of scrub typhus in resource limited countries. Scrub typhus IgM ELISA may be the best alternative test for routine use currently available in Nepal.

## Data Availability

All authors had full access to the data and materials. Data are available within this article. Detailed data is available from the corresponding author upon reasonable request.

## Abbreviation

ARRL: Australian Rickettsial Reference Laboratory
ELISA: Enzyme Linked Immunosorbent Assay
ICT: Immunochromatography
IFA: Immunofluorescence assay
IgM: Immunoglobulin M
